# *Chlamydomonas reinhardtii*: A Factory of Nutraceutical and Food Supplements for Human Health

**DOI:** 10.3390/molecules28031185

**Published:** 2023-01-25

**Authors:** Annalisa Masi, Francesca Leonelli, Viviana Scognamiglio, Giulia Gasperuzzo, Amina Antonacci, Michael A. Terzidis

**Affiliations:** 1 Institute of Crystallography, National Research Council, 00010 Montelibretti, Italy; 2 Department of Chemistry, University of Rome “Sapienza”, 00185 Rome, Italy; 3 Department of Nutritional Sciences and Dietetics, International Hellenic University, Sindos Campus, 57400 Thessaloniki, Greece

**Keywords:** *Chlamydomonas reinhardtii*, GRAS, high-value compounds, health, nutraceutical, biotechnologies

## Abstract

*Chlamydomonas reinhardtii* (*C. reinhardtii*) is one of the most well-studied microalgae organisms that revealed important information for the photosynthetic and metabolic processes of plants and eukaryotes. Numerous extensive studies have also underpinned its great potential as a biochemical factory, capable of producing various highly desired molecules with a direct impact on human health and longevity. Polysaccharides, lipids, functional proteins, pigments, hormones, vaccines, and antibodies are among the valuable biomolecules that are produced spontaneously or under well-defined conditions by *C. reinhardtii* and can be directly linked to human nutrition and diet. The aim of this review is to highlight the recent advances in the field focusing on the most relevant applications related to the production of important biomolecules for human health that are also linked with human nutrition and diet. The limitations and challenges are critically discussed along with the potential future applications of *C. reinhardtii* biomass and processed products in the field of nutraceuticals and food supplements. The increasing need for high-value and low-cost biomolecules produced in an environmentally and economy sustainable manner also underline the important role of *C. reinhardtii*.

## 1. Introduction

Microalgae represent a valuable source for economic production of commercially relevant high-value products (e.g., whole biomass, metabolites, recombinant proteins, etc.), in a more sustainable, environmentally friendly manner. In fact, the cultivation of microalgae may reduce the stress on intense resource demanding of terrestrial food crops. They also have higher photosynthetic efficiency and carbon mitigation potential, encouraging more sustainable and efficient production [1]. There are two main strategies used in industrial-scale production for algal biomass: i) open systems and ii) large closed systems that meet all the necessary photobioreactor criteria [2]. In terms of risk assessment and cost, the former increases the contamination risks (e.g., coliform bacteria and *E. coli*) due to a large surface of the biomass medium exposure [3], while the latter is costlier and provides much better control of the whole processes. The bioaccumulation of heavy metals represents another significant problem; thus, microalgae-growing media and water should always be carefully controlled [4]. It is worth noting that most of the processes used in microalgae production still struggles to reach a technology readiness level (TRL) that could demonstrate industrial viability, even if there is a significant number of new studies that provide important supplementary knowledge to that end (e.g., culture illumination influencing microalgal physiology, gene expression, and metabolism) [5]. For those cases that do not include whole-cell consumption, usually, the nutritional components of microalgae are extracted before being used. The various methods utilized to that end include steam explosion, ultrasonication, enzymatic disruption, and extraction with green solvents or CO_2_ under supercritical conditions [6]. The extraction processes are monitored for improving the efficiency and quality of the extract since it could be negatively impacted (e.g., oxidation). Regarding the microalgae extracts high in protein, they have been found to exhibit superior characteristics to those found in meat [7].

The pioneering keynote held by phycologist Ralph Lewin in 1992 at the Fifth International *Chlamydomonas* Conference has been a milestone and a predictive truth in *Chlamydomonas* history. The author described the photosynthetic microalgae, including morphological descriptions and genetics studies, as well as, coining its name *Chlamydomonas* (Greek etymology, χλαμύς (chlamys): a cloak; μονάς (monas): solitary) proposing the title of his address to be “*The cloaked one emerges from obscurity*” [8]. The advantages provided by a haploid system that gives access to all four products of meiosis, and due to the fact that the loss-of-function mutations can be immediately traduced in observable phenotypes with respect to diploid organisms, made the work on these systems highly desirable [9]. The easiness of growing the microalga *C. reinhardtii* and *Chlamydomonas eugametos* in the laboratory quickly and in large quantities (they boost a faster reproduction time (~ 8 h) in comparison with classic plant models) also played a crucial role

Taxonomic studies revealed the presence of more than 500 species belonging to the *Chlamydomonas* genus, with *C. reinhardtii* as one of the most well-studied [10]. The origin of *C. reinhardtii* was reported to derive from a single zygote isolated from a Massachusetts potato field in 1945 [11]. However, after decades of laboratory domestication (e.g., accumulation of mutations) their current genetic traits have become distant from the wild type, and they probably lost the capability to live in nature [10,12].

In the last decades, the research interest in this eukaryote organism has grown exponentially, focusing on the cell division, responses to excess light and the dissipation of light energy, metabolism, photosynthesis, cilia biogenesis, carbon-concentrating mechanisms, biosynthetic pathways, and chloroplast gene expressions. At the beginning of the 20th century, an important project related to the sequencing of the *Chlamydomonas* genome began, due to its strong potential in genetic engineering, which made it an excellent model system in DNA surgery and turning it into a model system dubbed “green yeast” [13,14]. Nevertheless, the preliminary gene models released were truncated or missing, mainly because of the limited technical limitations at the time.

For all these reasons, an iterative process of improvements housed at Phytozome, the Joint Genome Institute’s plant genomics portal, started and pushed *Chlamydomonas* into the “omics” age, thanks to the high-quality genomic data supported by RNA-Seq analysis and the ease-of-use organization on chromosomes [15,16].

The role of *C. reinhardtii* in developing genetic engineering protocols has been essential, and nowadays it is widely used to maximize the yields of bio-products, such as polyphenols, catechin, flavanols, glycosides, and phlorotannins, with intriguing applications in both medical and nutraceutical fields [17,18,19,20]. The abundance of noteworthy compounds produced by *C. reinhardtii* as primary or secondary metabolites made it an attractive source of carotenoids, chlorophylls lipids, polysaccharides, and recombinant proteins [5,21]. The recently discovered ability of *C. reinhardtii* to grow rapidly in fermenters has opened up the way for production at a commercial scale [22]. Moreover, the production of vaccines subunits, aiming to provide stable preparations, such as lyophilized microalgae pellets, could serve as alternative vaccinations in countries with health difficulties and they could also have a strong impact on the reduction in production costs, due to easiness in production, handling, and vaccine administration [23,24,25]. The notice (No. 773) issued by the Food and Drug Administration (FDA) recognizing *C. reinhardtii* as a generally regarded as safe (GRAS) organism unveiled new possibilities for further utilization of *C. reinhardtii*.

Recent studies have highlighted the positive health outcomes of algal compounds inserted into the human diet. In particular, microalgae biomass and extracted compounds have been tested, providing promising results in terms of potential reduction in cancer incidence, prevention of diseases, and control of inflammation and obesity [26,27,28,29,30].

Clinical studies on effects related to the consumption of *C. reinhardtii* whole cells by human volunteers have been carried out for assessing the impacts on gastrointestinal health and microbiota, showing that the intake of *C. reinhardtii* cells is able to promote the microbiota eubiosis, reducing the imbalances and improving the general health of the intestine, even in conditions of induced colitis stress [31].

Another significant role of *C. reinhardtii* in human health concerns antibiotic resistance, as it has been recently defined by the World Health Organization (WHO) as the most serious warning to the health of the global population. The ability of *C. reinhardtii* to synthesize several metabolites exhibiting strong antibiotic activity to counteract numerous pathogenic microorganisms may prove to be an important asset [32]. Among those metabolites, the sulfur polysaccharides are believed to be some of the most promising metabolites, inhibiting the formation and promoting the elimination of bacterial biofilms which trigger strong virulence in many foods and represent a serious problem in hospital environments [33,34].

Achieving better high-value nutrients and recombinant protein content in *C. reinhardtii* has constantly been the aim of many studies utilizing genetic and metabolic engineering tools; for example, toward the expression of human selenoprotein, production of bovine lactoferricin, increment of PUFA levels, fatty acids (FAs), anti-p57 antibodies, and carotenoid pigments [35,36,37,38,39,40,41].

The aim of this review is to describe the role of *C. reinhardtii* in supporting human health by the production of various high-value compounds (e.g., polysaccharides, lipids, proteins, and pigments) that can be directly used as nutraceuticals or food supplements. We also seek to highlight the biotechnological advances in the field by giving special attention to the most recent applications. Lastly, we underline the still unexploited potential of this system and the challenging issues still pending to be tackled.

## 2. Bio-production

### 2.1. Polysaccharides

Polysaccharides are macromolecules consisting of 10 or more monosaccharide units connected to each other by glycosidic bonds.

According to their composition, the structure and, therefore, the physicochemical characteristics of the polysaccharides can be different properties, such as gelling, texturizing, thickening, and stabilizing [42,43]. Based on these aspects, polysaccharides find applications in various fields ranging from the food industry to the medical and pharmaceutical sectors, to biotechnological procedures [44]. Polysaccharides play significant roles as immunomodulators, antioxidants, antitumor drugs, anticoagulants, and antiviral agents. Algal cells are an abundant resource of these macromolecules [45].

In particular, *Chlamydomonas* species, depending on the strain, are known to produce different families of carbohydrates, namely exopolysaccharides (EPSs) and sulfated polysaccharides (SPs). EPSs are released by microalgae or encapsulated in the cell walls of microorganisms. These polysaccharide excretions have, among other functions, that of protecting microalgal cells from harmful agents and/or environmental stress [46,47]. Moreover, they have many important technological and physiological properties of high interest in the food industry safely; they are environmentally safe and usually biocompatible. Any variety of the same species is able to supply different EPSs because this production is not species-specific [43]. The chemical analysis of EPSs secreted by several *Chlamydomonas* strains reveals the presence of six to nine monomers, with the main representatives being glucose, galactose, fucose, rhamnose and arabinose (Table 1).

EPS production and its physicochemical properties can be influenced by growth conditions such as medium composition, salinity, nutrient availability, light exposure (photon flux and wavelength), pH, and temperature. Abiotic stressors cause algal cells to secrete polysaccharides [46]. For example, several studies of *C. reinhardtii* have shown that growing the microalga under S-starvation induces a 10- to 15-fold increase in carbohydrate accumulation [51,52]. For all these reasons, the studies on EPS production from *Chlamydomonas* microalgae represent an intriguing goal for the research.

Regarding the SPs, they are polymers with anionic nature composed of one or more monosaccharides substituted with a sulfate group in different positions, exhibiting significantly useful biological properties [53,54,55,56] such as anticoagulant [57,58,59], anti-viral [60,61], antioxidant [62,63,64,65], anticancer [60,66,67,68,69], antithrombotic [70], antimicrobial [71], anti-obesity [72,73,74], and anti-inflammatory activities [75,76,77]. Green algae biosynthesize diverse types of SPs with different carbohydrate residues such as xyloarabinogalactans, glucuronoxylorhamnans, glucuronoxylorhamnogalactans, etc [55]. Numerous studies have demonstrated that the composition of SPs is strongly correlated with their bioactivity, and the production of different SPs from diverse algae. For example, carrageenans and agarans are biosynthesized from red algae, while brown algae produce laminarin, alginates, and fucoidan.

Recently, is was found that SPs possess significant antibacterial activity, even against multiresistant bacteria [32]. Several studies have focused on the possible mechanism which allows these metabolites to prevent antibacterial activity from taking place. The mechanism proposed is based on the binding of the polysaccharides to the cell wall, cytoplasmic membrane, and DNA of the bacterium. These interactions cause dramatic changes in the bacterial cell, such as membrane modification, resulting in protein loss and bacterial DNA damage [78]. In particular, SPs from *C. reinhardtii* exhibited significant antibacterial activities toward opportunistic pathogens such as *Streptococcus* sp., *Bacillus subtilis*, *Neisseria mucosa,* and *Escherichia coli*. Interestingly, at concentrations of 4–8 mg/mL, the extracted SPs inhibited at 100% the biofilm formation of all bacteria [33]. Moreover, other noteworthy properties of SPs from green chlorophyte *C. reinhardtii* have been reported, including efficient antioxidant and anticancer activities toward different cancer cell lines (e.g., HepG2, HeLa, melanoma B16, and MCF-7), although the molecular mechanism still remains unclear [34]. SPs have also shown promising anti-neurodegenerative activities by both inhibiting the α-Synuclein (α-Syn) fibrillation process and dissolving preformed fibrils [33,79].

The production of EPSs and SPs from *C. reinhardtii* can be considered economically sustainable, but still many efforts are required to be made to achieve optimal growth conditions, to identify the best-performing strains in the production yield, and finally, to deepen our understanding of the molecular mechanisms of these compounds related to their biocidal and antitumor activities.

### 2.2. Lipids

Lipids are organic compounds widely diffused in nature and represent one of the four main classes of organic compounds of biological interest, together with carbohydrates, proteins, and nucleic acids. Lipids are identified on the basis of their common solubility properties. They are insoluble in water (therefore, they are defined as hydrophobic), while they are soluble in organic solvents such as diethyl ether or acetone, alcohols, and hydrocarbons. These molecules perform fundamental biological functions, they are the principal components of cell membranes (e.g., galactoglycerolipids, phosphoglycerolipids, sphingolipids, and sterols), they are responsible for energy storage (triacylglycerols or TAGs) and for signaling in defense responses (e.g., phosphoinositides and oxylipins) [80]. Furthermore, they find broad applications. Among the main ones, they are used as essential nutrients in human and animal nutrition, important raw materials for industry, potential renewable energy resources, nutraceuticals, and food supplements [81].

The potential of lipids present in *C. reinhardtii* for biofuels and bio-products production is well-known and previously reviewed elsewhere [82,83]. In this review, we seek to point out an additional value of these compounds related to their nutritional capacity.

Microalgae are among the main producers of very long-chain omega-3 and omega-6 polyunsaturated fatty acids (PUFA). These compounds play critical roles both as nutrients in the human diet and are used in the medical field [84,85]. Furthermore, some of these are considered essential fatty acids because they cannot be biosynthesized by humans, but must be taken through the diet. Moreover, the potential health benefits of the ω-3PUFAs, namely alpha-linolenic acid (ALA), docosapentaenoic acid (DHA), and eicosapentaenoic acid (EPA), on neurological disorders, inflammatory disease, cardiovascular events, and even some cancers, have been well-investigated [86,87,88,89]. Microalgae can be induced to affect their lipid metabolism if subjected to stressful conditions. In particular, stress conditions can be identified as unfavorable environmental factors, such as illumination conditions (wavelength, duration, and intensity), carbon dioxide levels, temperature, nutrient starvation, heavy metal stress, pH, and saline stress, or a combination of the latter factors. For example, if all the elements necessary for growth are not available, microalgae cells can divide rapidly, mainly synthesizing membrane lipids, in particular chloroplast lipids. Moreover, extreme temperatures are reported to be related to changes in the composition and the ratio between membrane lipids in order to reach a phase of adaptation of the cell to the new conditions. Furthermore, light intensity, spectral quality, and the photoperiod are known to affect the metabolism of microalgal lipids and their lipid composition. Indeed, in the case of high luminous fluxes, microalgae can reduce the size of chloroplasts and induce deep rearrangements in granae and intergranal lamellae, which is in agreement with the reduction in the proportion of membrane lipids [90]. In the last decades, many efforts have been dedicated to finding the optimized stress conditions to be applied to microalgae cultures for reducing cultivation costs, maximizing the accumulation of desirable compounds, and preventing contamination by undesirable organisms. Therefore, this approach represents a sustainable and cost-effective strategy to increase lipid production in microalgae [91].

The percentage of lipids contained in microalgae is between 20 and 50% of dry weight; the amount depends on the algal strain and growth conditions [92]. In particular, *C. reinhardtii* exhibits about 20% of lipids [93], which are composed of saturated, monounsaturated, or PUFAs with a length between 16 and 20 carbons. The most abundant are 16:0, 16:4^4Z,7Z,10Z,13Z^, 18:1^9Z^, and 18:3^9Z,12Z,15Z^ (Table 2) [20].

In their study, Darwish and co-workers [20] compared the lipid content in *C. reinhardtii* with that of *Chlorella* and *Spirulina*, which are considered to be important principle active ingredients in the production of health food supplements. The authors found that the amounts of FAs, particularly omega-3 FAs, contained in *C. reinhardtii* are superior to the reference species in both quality and quantity. For example, as shown in Table 2, the content of ALA (C18:3 n-3) in *C. reinhardtii* is considerably higher than that of *Spirulina* and *Chlorella*. Moreover, computational tools assisted by input from experimental data revealed that tailor-made cultivation strategies relying on optimal nutrient composition could yield significant increases of +270% in the starch and +74% in the lipid content in *C. reinhardtii*, opening new avenues for manipulation of the nutrient composition [94]. It is important to underline the determining role of ALA for humans. It is the starting material from which EPA and DHA derivatives can be synthesized [95]; therefore, the dietary intake of this precursor could lead to the formation of DHA and EPA with their proven beneficial effects. However, it must be specified that the transformation of ALA into EPA and DHA in humans is not quantitative, but occurs in various percentages, depending on the quantity of omega-6 present. Specifically, the conversion in EPA is about 8%, while in DHA is about 0.1%; therefore, the intake of FAs only with *C. reinhardtii* would not be sufficient to meet the daily requirement but would have to be integrated with a balanced diet.

An important aspect to consider is that *Chlamydomonas*, compared to other microalgae species, is fully characterized. Its genome, in fact, is completely sequenced allowing the development of sophisticated genetic engineering protocols [13,96,97,98].

In recent years, there has been considerable interest in using microalgal lipids in the food, chemical, pharmaceutical, and cosmetic industries. In the fuel industry, algae-based biofuels have emerged as clean, nature-friendly, and cost-effective solutions compared to other fuels [99]. In cosmetic formulations, lipids and their derivatives are one of the main ingredients [100]. Algae as a food has been explored for different applications such as in the production of human food, as fodder for fish and farm animals, and as food supplements [101]. Although microalgae are viable sources for bioenergy and biopharmaceuticals in general, some limitations and challenges remain, which need to be overcome to upgrade the technology from the pilot stage to the industrial level.

### 2.3. Recombinant Proteins for Therapeutics

Therapeutic proteins are genetically modified human proteins used for pharmaceutical purposes. They have been shown to be highly effective in vivo and are invaluable tools for disease treatment. In fact, protein therapy allows for a targeted and personalized therapeutic approach based on the individual’s specific deficiencies.

Photosynthetic microalgae provide a broad spectrum of complex proteins and, compared to other systems, such as bacterial, mammalian, yeast, viral, insect, higher plants, etc., they offer significant advantages. In particular, they are cheap, scalable, and safe, allowing uses aiming to a potential oral therapeutic delivery [102]. Furthermore, environmental contamination of therapeutic proteins can be reduced as algae can be grown in full containment.

Algal chloroplasts, compared to cytoplasm, are considered excellent targets for synthetic biology approaches due to the relatively small size of their genomes (plastome, constituted by ~100–200 genes, encoding for core elements of the photosynthetic complexes, and transcription/translation factors of the plastidial apparatus) completely separated from the nuclear genome. Moreover, chloroplasts are believed to be a preferential target for the expression of recombinant proteins in comparison to other microbial, mammalian, or plant-based systems for their specific features. In particular, they boast the possibility of a high precise and specific insertion of heterologous DNA via homologous recombination, avoiding gene silencing problems. Moreover, the recombinant product’s efficient folding and accumulation can be achieved thanks to the chaperones and disulfide isomerase activities, which are able to support their native states. The procedures for product purification in C. reinhardtii can be significantly simplified thanks to its GRAS status and the absence of harmful viral, prion, or endotoxin contaminants [103,104].

In particular chloroplasts of microalgae are of relevant interest for recombinant protein expression; in fact, large groups of protein complexes are produced by these organelles including full-length monoclonal antibodies [105], chimeric anti-cancer immunotoxins [106], and other therapeutic proteins, such as erythropoietin, human fibronectin, interferon, grow factor, etc. [107]. *C. reinhardtii* chloroplasts constitute about 70% of the cell volume and, potentially, they represent a source of new therapeutic proteins of rapid development and low cost, thanks to the synthesis of plastidial proteins such as disulfide isomerases, chaperones [108], and peptidyl propylisomerases able to form disulfide bridges [109].

*C. reinhardtii* is a good bioreactor to produce important recombinant proteins [110,111]. The studies conducted by Almaraz-Delgado et al. fit into this context. They indeed identified several therapeutic proteins, including antibodies, enzymes, therapeutic proteins, viral proteins, etc., produced in the chloroplast of *C. reinhardtii* (see Table 3). Thus, the ongoing research efforts aim to optimize the growth conditions [112] and the processing of microalgae [113] in order to increase the production of recombinant proteins [112].

**Figure 1:**
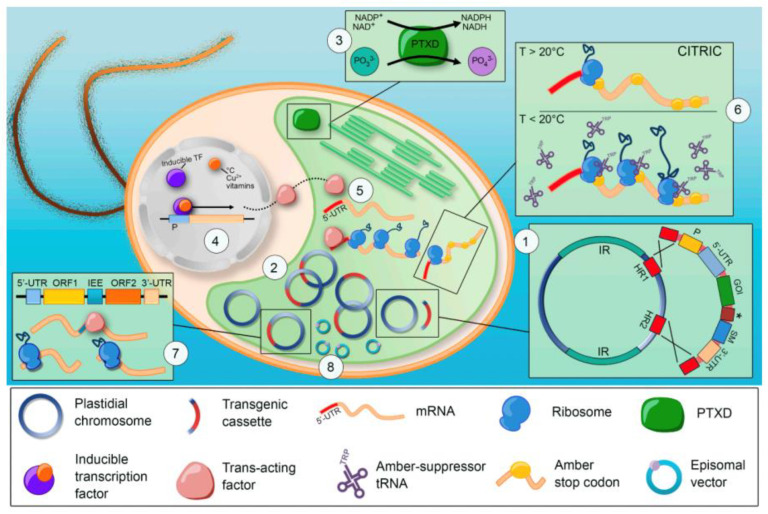
Chloroplast transformation in *C. reinhardtii.* Reprinted from ref. [103].

In addition, the biotechnological industry benefits from the high-value molecules produced by genetically engineered *C. reinhardtii* chloroplasts (Figure 1).

Almaraz-Delgado et al. performed the *C. reinhardtii* chloroplast modification utilizing a particle bombardment device, where the gold particles used for bombardment are covered with a plasmid transporting the genes of interest [114]. When these particles, accelerated by helium gas, reach and penetrate the cells, homologous recombination occurred and the regions present in the plasmid were integrated with those present in the chloroplast genome (CpDNA). This technology has proven to be the best option for the preparation of certain proteins, otherwise difficult to obtain, such as immunotoxins [106,125], non-glycosylated antigens [24,124], and antibodies [116].

The studies conducted by Rasala and Mayfield [126] focused on the preparation of human therapeutic proteins from *C. reinhardtii*. Indeed, they modified the CpDNA by inserting in it seven unrelated genes that encode existing or new human therapeutic proteins, highlighting that four of these genes promoted the accumulation of bioactive proteins at sufficient levels for commercial production.

Recently, Pang and co-workers [127] explored the possibility to express the human lactoferrin (hLF) in *C. reinhardtii*. Lactoferrin is an iron-binding glycoprotein found in nature that possesses different and interesting biological functions, such as antibacterial, antiviral, and antitumor activity, etc. The recombinant hLF, obtained from *C. reinhardtii* algae, showed notable antibacterial activity and low toxicity to mice. Moreover, in comparison with commercial hLF expressed in rice, the recombinant hLF produced by algae displayed comparable or moderately lower stability under various experimental conditions (temperature and pH) [127], indicating that expression of hLF in *C. reinhardtii* is accessible and thus opening the way to the possible preparation and administration of lactoferrin directly using an eatable *Chlamydomonas.* Although the potential of *C. reinhardtii* as a vehicle to synthesize pharmaceuticals and biotech products is clear [111], the extreme randomness of the locus of exogenous gene integration, due to the lack of homologous recombination in the nucleus, unlike the plastid zones, represents a great limitation. In fact, the insertion of exogenous DNA at random loci can result in gene deletion, recombination, and translocation near the integration site. This is one of the reasons why the transformation in the nucleus of an exogenous protein in *C. reinhardtii* is not yet fully established. In this context, the research aims to understand the gene expression mechanism in *Chlamydomonas* by developing and optimizing potent transcription and translation elements, as well as developing powerful genetic tools to obtain stable and working nuclear transformation to enhance protein expression efficiency.

### 2.4. Pigments

Several species of microalgae show the natural attitude to produce pigments essential for their photosynthetic physiology (e.g., *β*-carotene, fucoxanthin, phycoerythrin, and lutein). These are secondary metabolites with the main function to sense light and control its intensity to support the correct growth of microalgae cells and their rapid responses to the environment [128].

This group of bio-active compounds has been deeply investigated for its antihypertensive, anticarcinogenic, and antioxidative features, as well as for its promising health benefits upon consumption, as dietary and nutraceutical supplements in human diet toward a better quality of life from a clinical point of view [129]. Moreover, several studies highlighted that the pigment content in microalgae cells is higher in comparison with different plants and floral species [130].

The use of *C. reinhardtii* as a model system for the synthesis of photosynthetic pigments represents a fascinating reality, in particular for the possibility to enhance their productivity through variations in growth conditions and genetic changes. Recently, Rathod and colleagues explored the possibility of using *C. reinhardtti* as a technological platform in an interesting molecular biology experiment [131]. In detail, they genetically manipulated *C. reinhardtii* to obtain a functional expression of native phytoene-*β*-carotene synthase from *X. dendrorhous*, encoding a bifunctional enzyme and obtaining an enhanced flux of carotenoid pathway; that is, *β*-carotene and lutein accumulation. Moreover, the boost of *β*-carotene (increased by 84 %; *p* < 0.01) and lutein (increased by 75%; *p* < 0.01) led to a distinctive pale yellowish-light phenotype of novel mutant, contrary to the dark green WT [131]. Another fascinating experimentation on *C. reinhardtii* cells has been conducted by Song and colleagues aiming to produce highly purified zeaxanthin from algal mutants for personalized treatment and pharmaceutical applications. Thus, they generated a double knockout mutant by the clustered regularly interspaced short palindromic repeat CRISPR-associated protein, the CRISPR-Cas9 ribonucleoprotein-mediated knock-in system. They edited the lycopene epsilon cyclase gene and the zeaxanthin epoxidase gene, obtaining a double mutant with a 60 % higher zeaxanthin yield (5.24 mg L^−1^) and content (7.28 mg g^−1^) than that of the parental line after 3 days of cultivation [132].

Another carotenoid biosynthesis enhancing in *C. reinhardtii* has been obtained by Cordero and colleagues with a nuclear transformation of the phytoene synthase gene isolated from *Chlorella zofingiensis*. The authors showed an increase in the expression levels of this gene, as well as a higher content of violaxanthin (2.0-fold) and lutein (2.2-fold) with respect to the untransformed cells [133]. In the context of pigment production, a crucial aspect relates to environmental conditions, such as the wavelength within the electromagnetic spectrum and temperature. This was well-documented by Zhao and colleagues, who demonstrated an optimal growth of *C. reinhardtii* under white light irradiation at 35 °C, while mixing white light and blue light at a 3:1 ratio and a temperature of 20–25 °C, which were found to be the best conditions, leading to the highest lutein accumulation (lutein content of 4.24 mg g^−1^ and productivity of 3.25 mg/L/day) [134].

Following the studies regarding the relationship between autophagy and carotenoids in microalgae, Tran and colleagues [135] recently proposed an innovative approach to boost the synthesis of carotenoids by the regulation of autophagy and carotenoid biosynthesis pathways in *C. reinhardtii*. The processes of autophagy represent a self-recycling mechanism of eukaryotes, and in particular, in the microalgae cells, it is responsible for senescent or oxidized cellular components cleaning that could lead to an oxidative chain reaction and trigger cell death. Hence, it was exploited the role of autophagy silencing the ATG1 and ATG8 genes using artificial microRNA, which in turn reduced the mRNA expression of ATG1 and ATG8 by 84.4% and 74.3%, respectively. This gene expression level decrease led to a 2.34-fold increase in the amount of *β*-carotene content (23.75 mg g^−1^).

The interest in using *C. rehinardtii* cells as bioreactors for the production of pigments is evident and contains a high potential in the medical and nutraceutical field. Moreover, the possibility of expanding these technologies to other species of algae could be interesting in the resolution of some current limitations related to the use of *C. reinhardtii* (enhanced capability to grow in open ponds or photobioreactors, faster growth, as well as little susceptibility to contamination). This could represent a new push toward the development of novel genetic tools and transformation methods in microalgae.

## 3. Nutritional Aspects: Linking with Human Diet and Related Food Products

Studies examining the consumption of *C. reinhardtii* by *Daphinia* species date back to 1968 [136,137,138,139,140]. *C. reinhardtii* has been found in the epicenter of numerous studies to date as a model [8,139,140]; however, it was not until very recently, in 2018, that the dried biomass of *C. reinhardtii* was considered food or a food ingredient. It is one of the very few microalgae (wild type strain—THN 6) that has already received the GRAS status from the FDA in the USA as a nutritive ingredient in food for replacing dietary proteins in the population of aged >2 years [141].

The absence of known bioactive or toxic compounds among those produced by *C. reinhardtii* and its composition, as depicted in Table 4, initially suggested physiological digestion, absorption, distribution, metabolism, and excretion processes similar to other foodstuffs consumed by humans. The latter hypothesis was later confirmed by in vitro and in vivo experiments involving rats that showed no adverse effects. The studies were focused on the toxicological evaluation of the dried biomass of *C. reinhardtii* wild type strain (THN 6) and particularly the genotoxic potential and the repeated-dose oral toxicity by application of international accepted standards according to the following tests: (i) bacterial reverse mutation; (ii) in vitro mammalian chromosomal aberration; (iii) in vivo mammalian micronucleus; and (iv) a 28-day in vivo repeated-dose oral toxicity study involving rats [142]. For estimating the dietary exposure for the model studies, it was initially calculated that the usual lifetime protein intake for the population in the USA is 114.8 g/day and body weight (bw) is 2.092 g/kg bw/day. This number was then adjusted to fit into a more realistic scenario on the hypothesis that the dried algal biomass could potentially replace up to 10% of the dietary protein consumed; thus, 16.4–38.3 g/day or 0.299–0.697 g/bw/day, which is equivalent to 11.5 g protein/day or 0.209 g/protein/bw/day, respectively [141].

To our knowledge, there are only very few toxicological studies employing mammals (i.e., rats and mice) and just a very recent one, in 2020, involving human volunteers [31,142]. The results of the latter 30-day dietary study, which was focused on the impact on gastrointestinal health, showed that the consumption of 1–5 g/day freeze-dried algal biomass led to a notably better gastrointestinal function, while the gut microbiome composition is not affected significantly. Regarding the organoleptic characteristics of the dried biomass, most of the participants in the study found the powder’s appearance to range from indifferent to very appealing, while the flavor/odor and taste had a normal distribution from very unappealing to very appealing, on a 1–5 scale. The study also highlighted that the algal biomass consumption mitigated weight loss in a dextran sulfate sodium colitis model in mice, while in humans with high-frequency gastrointestinal symptoms, it reduced those associated with irritable bowel syndrome and improved the quality of stools. These results indicated the action of unknown bioactive compounds present in algal biomass and/or potentially a shift in gene expression of the gut bacteria of the consumer. It should be noted also that some of the authors involved in the studies declared competing interests and the *C. reinhardtii* biomass was provided by Triton Algae Innovations, which also funded part of the research [31,141].

The standard specifications of the food grade product, extracted by the application submitted to the FDA, are listed in Table 4, along with data reported from two different batches, namely TAI-1215-01 and TAI-0316-01. The % of dry biomass composition of *C. reinhardtii* on the major substituents, such as proteins, carbohydrates, and lipids has been calculated to be 22–48%, 15–50%, and 18–29%, respectively [142,143,144,145].

Except for the wild type THN 6, recently other *C. reinhardtii* strains have been considered for human consumption. The increasing need for new and novel sustainable strategies for the development of alternative plant-based products that could substitute meat products pinpointed the value of heme-containing proteins that could induce a meat-like color and a “meaty” flavor to the products added. To that end, some engineered *C. reinhardtii* strains overexpressing iron-complexed proteins, such as hemoglobin, myoglobin, leghemoglobin, and protoporphyrine IX (PPIX), are proposed in recent patent applications [147,148,149,150,151,152] as very attractive alternatives to other related examples of iron-complexed proteins; for example, soy leghemoglobin in genetically engineered yeast of *Pichia pastoris* [153,154]. The selected *C. reinhardtii* strains are unable to synthesize chlorophyll and they are light sensitive, exhibiting red, brown, orange, or a variation of these colors. To that end, for example, the overexpression and accumulation of protoporphyrine IX were found to be important; thus, the heme biosynthesis pathway was found in the epicenter of the investigation along with the development of purification and algae/composition enrichment procedures [151]. The edible compositions proposed for preparing ingredients of finished products, such as meat or fish analogs, e.g., heme-enriched “meatless” burgers, tuna, and plant-based burgers, including whole-cell algal biomass, fractionated algal biomass enriched in PPIX and protein, or extracellular fraction of the algae culture. The heme-enriched algae, containing 4.5% protoporphyrine IX and 24.4% protein, was used in 0.01–5 % *w*/*w* compositions, inducing a significant change in the color of the final product [150,151].

## 4. *C. reinhardtii* Molecular Genetic Toolkit: Is an Effective Road for the Market?

**Figure 2:**
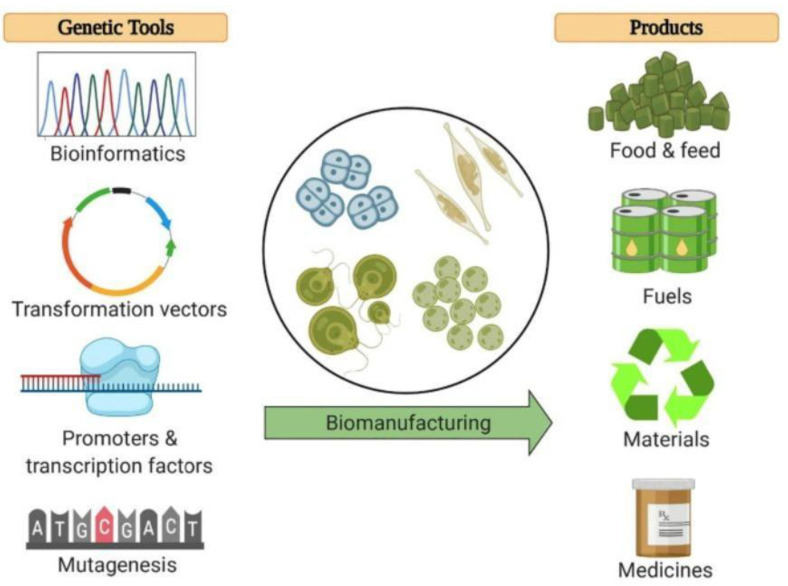
Genetic tools to develop microalgae as a platform for the biomanufacturing of commercial products. Bioinformatic algorithms are used to analyze algal genome sequences, resulting in codon optimization and motif discovery techniques that allow the design of strong expression vectors for the genetic transformation of algae strains. Regulatory elements, such as promoters and transcription factors, allow recombinant gene expression and manipulation of metabolic pathways to obtain products of interest. Random mutagenesis and genome shuffling can further drive the algal production strains toward desired phenotypes. These tools are being utilized to explore the industrial production of foods, fuels, materials, and medicines from microalgae. Reprinted with permission from ref. [155]. Copyright 2021 Elsevier.

One of the main challenges in the bioproducts made from *C. reinhardtii* is the evolution from a small volume of the laboratory to a large industrial scale, due to the costs of maintenance and protection from possible contamination by other microorganisms. Interesting insights come from the manipulation of algal strains to improve the production of valuable compounds, and, in this context, the biotechnologies related to genome editing are providing valuable support. Indeed, it is undeniable that microalgae are a promising genetically manipulated platform for achieving bio-products in a sustainable and quick way with excellent yields. The strong potential lies in their expression vectors, regulatory elements (e.g., promoters and transcription factors), and the availability of several transformation methods (Figure 2) [155].

**Figure 3:**
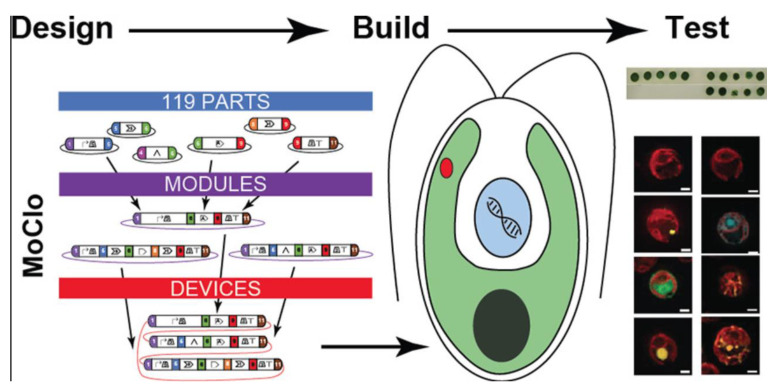
MoClo for the green microalga *C. reinhardtii*. It is based on Golden Gate Cloning with standard syntax and comprises 119 openly distributed genetic parts, most of which have been functionally validated in several strains. Reprinted with permission from ref. [156]. Copyright 2018 ACS Publications.

Crozet and colleagues [156] established a flexible cloning toolkit (MoClo) for *C. reinhardtii* based on Golden Gate Cloning with standard syntax, inclusive of 119 openly distributed genetic parts. The latter provided a rapid method to design engineered cells through promoters, reporters, UTRs, tags, terminators, antibiotic resistance genes, and introns cloned in different locations with extreme modularity (Figure 3).

*C. reinhardtii* has been further investigated to validate the amounts of nuclear heterologous gene expression by computational analysis and to overcome this strong limitation in a commercial setting. Different sequence optimization algorithms have been analyzed, in particular, eight distinctive sequence encodings for a synthetic ferredoxin-hydrogenase enzyme were achieved and utilized as a gene reporter. The nuclear transformation was carried out in line with previous studies in silico and the collected data showed the significance of proper codon optimization to obtain high transgene expression [157].

Likewise, engineering recombinant proteins in microalgae is showing great attention thanks to the insertion of the exogenous gene encoding for protein, enabling it to express desired compounds at high levels and target to specific algal cells in the subcellular region [158,159]. However, in this case, some issues arise, particularly the lack of the desired protein accumulation is usually hampered by the transcriptional or post-transcriptional levels. For all these reasons, the interest in the identification of promoters able to drive good production of the target protein is very high, such as in *C. reinhardtii,* the promoter of heat shock protein 70 (HSP70) [160]. A recent application of this promoter [161] showed an increase in the productivity of biomass in *C. reinhardtii,* overcoming the problem of high thermal dissipation in case of high light intensity. In particular, the light use efficiency of microalga was improved, introducing an LHCSR3 gene under the control of HSP70/RUBISCO small chain 2 promoters in the mutant *npq4 lhcsr1*, which is the knockout for all LHCSR genes. This resulted in a low expression of LHCSR3 and a lowering of the photoprotective mechanism, and also in improved photosynthetic effectiveness, as well as in elevated biomass production.

The recombinant proteins importance in *C. reinhardtii* has been widely documented in the biopharmaceutical field, making growth factors, vaccines, cytokines, antibodies, hormones, antibiotics, thrombolytic agents, enzymes, and immune signaling proteins [127,162,163,164]. The choice of this microalgae is derived from its eukaryotic genetics, which makes it possible to overcome some of the hitches encountered with other organisms (e.g., bacteria and yeast), such as costs, product effectiveness, low yields, contamination, and immunogenic effects [165,166].

Concerning the synthesis of bio-products with intriguing effects on human health, several studies highlighted also the power of metabolic engineering to overexpress or knock out pivotal gene encoding for enzymes involved in FA, isoprenoid, or carotenoid pathways. A noteworthy example has been recently reported in the literature, demonstrating the possibility of enabling *C. reinhardtii* to generate high-value ketarotenoids (for example, canthaxanthin and astaxanthin). In fact, the enzyme *β-carotene ketolase* was found in the microalga genome, but physiologically there was little expressed. An overexpression of this enzyme was achieved thanks to the codon optimization and systematic incorporation of the first intron of the rubisco small subunit II gene. This experiment design led to a strong production of ketocharotenoids, consequently triggering the change in the cell color from green to reddish-brown, as well as motivating a competitive production with *Haematococcus pluvialis*, presently the primary organism in the production of industrial astaxanthin [167].

The above-described examples highlight the recent interest in creating or optimizing transgenic strains and, hence, improve the industrial production of various compounds [101]. Several genome editing approaches are available, such as zinc finger nucleases (ZFNs), RNA/DNA gene repair, transcriptional activator-like effector nucleases (TALENs), and CRISPR-Cas [168,169]. Each technique has its pros and weaknesses, but lately, CRISPR-Cas9 is enjoying great success in many model organisms owing to its versatility, accessibility, and precision. Concerning the CRISPR-Cas system-mediated transformation for *C. reinhardtii,* various studies have been reported, but several issues remain to be solved resulting in low success. In particular, the main problems arise from repair mechanisms and off-target effects, which make genetically unstable microalgae and conventional Cas9 and single guide RNA (sgRNA) genes inefficient [170]. Additionally, novel transformation methods for *C. reinhardtii* based on DNA-free CRISPR systems and visual phenotypic observation have been reported [171]. A noteworthy example has been proposed by Baek and colleagues for generating a *C. reinhardtii* ZEP knock-out mutant from the CC-4349 strain by direct delivery of ribonucleoproteins without the transport of foreign DNA. This study represents an uncommon example of lutein and zeaxanthin production for commercial application as a feed supplement to produce macular pigment-enriched eggs, limiting the off-target effects [172].

## 5. Conclusions and Future Perspectives

To date, *C. reinhardtii* has been mostly used on a lab scale as a model organism in investigations focused on the biological photosynthetic and metabolic processes of plants and eukaryotes [36]. Its synthetic biology performance underpins a high potential for using it as a biochemical factory for the production of various valuable biomolecules, such as proteins, hormones, vaccines, antibodies, etc. Regarding the genetic manipulation of *C. reinhardtii*, important achievements have been already reported, such as the reduction in costs and the standardization of molecular toolkits able to offer the possibility of using a modular system with standardized parts (promoter, coding sequence, and regulatory elements) easily inserted, interchanged, or removed. The emerging and successful use of CRISPR/Cas techniques made possible accurate genome editing by solving some of the drawbacks currently still present in the genetic manipulation of *C. reinhardtii* (e.g., random integration of the genome, problems of positional effects, or the epigenetic gene silencing). Hence, thanks to the rapid pace of technological progress, it is possible that *C. reinhardtii* will soon become a new platform for industrial biotechnology, as has already happened in the past for other model systems (e.g., *E. coli* and *S. cerevisiae*). Significant advances have been also described in modifying the metabolic pathways of carotenoids with consequent accumulation of *β*-carotene, zeaxanthin, and astaxanthin, as well as in the production of recombinant proteins.

Several foodstuffs of high consumption still lack several important components, thus limiting their nutritional value. Most infant formulas, for example, lack or contain a much lower level of “functional proteins” necessary for infant development, i.e., osteopontin, sCD14, lysozyme, lactoperoxidase, immunoglobin A, and superoxide dismutase [37,38,39,148]. Due to the rapid advances in the field of *C. reinhardtii* biotechnology, including precise and programmable genome editing and application of new tools for transgene expression, cloning, and transformation [173,174], the realization of robust scalable solutions is expected to come soon, reaching in efficiency the processes commonly used in traditional crops [175].

The exploitation of the research outcomes in the field of algae recombinant products has been mainly limited until now and one reason is the fact that a viable large-scale production process is still in its infancy. This has also limited the recombinant products produced by *C. reinhardtii* that could potentially be found in the market since the preliminary laboratory studies have provided important proof-of-concept results [175,176,177,178,179,180]. The ability of the genetically engineered *C. reinhardtii* to transform phosphite to phosphate and to grow successfully in harsh environments, such as wastewater, where bacteria and other biological contaminants are found at high populations and concentrations, respectively, is a highly desirable characteristic for achieving high-quality large-scale production [181]. *C. reinhardtii* strains developed by genome shuffling were found to exhibit good tolerance in high salinity [182]. Recently, some of the limitations seem to be addressed by the announcement of a new validated commercially viable production process, leading to an increment of *C. reinhardtii* production to >20 g/L/day, with estimations of the cost dropping about 5-fold after successful scale-up [148].

Apart from the induced synthesis of bioactive components and their nutritional values, the photosynthetic H_2_ evolution from *C. reinhardtii* was found to increase 7-fold when grown in advanced solid-state fermentation wastewater compared to the cells grown in a tris-acetate-phosphate medium, thus opening up a new avenue for uses also in waste treatment [183].

The preliminary positive results from a murine model of acute colitis studies and gastrointestinal health in humans [31] have increased the expectations [184] that algae could reveal new molecules of high interest for further improvement of the human diet [185,186]. Overall, *C. reinhardtii* is expected to play a huge role as a delivery platform in the pharmaceutical/nutraceutical industry in the future [151,172,187].

The sensory features, such as aroma/flavor and texture, are also important factors to be considered before being accepted by consumers. We should note here that some of us are currently working on methods for improving the aroma and investigating the stability of the *C. reinhardtii* dried biomass under controlled environmental conditions.

## 6. Methodology

The literature search was performed by using five electronic databases, namely, PubMed, Scopus, Google Scholar, Google Patents, and EPO—espacenet, for identifying the related peer-reviewed journal articles, edited academic books, and patents. We have used keywords such as *Chlamydomonas reinhardtii*, *C. reinhardtii*, GRAS, high value compounds, health, nutraceutical, food and biotechnologies, and combinations of these. We also searched the references and citations of our search results to identify related articles and patents. This review summarizes the results published until December 2022.

## Figures and Tables

**Table 1:** Sugar monomers of polysaccharides in *Chlamydomonas* microalgae where **Ara** arabinose, **Fuc** fucose, **Gal** galctose, *Glu* glucose, *Man* mannose, **Rha** rhamnose, *Rib* ribose, **Xyl** xylose, **UA** uronic acids. Adapted from [48].

Species	Sugar Monomers (in Alphabetical Order)	Ref.
*C. mexicana* (or *C. oblonga*) ^1^	Ara, Fuc,^2^ Gal, Glu,^2^ Man, Rha, Rib, Xyl, UA	[49]
*C. reinhardtii*	Ara, Gal, Glu,^2^ Man, Rha	[50]
*C. sajao* (or *Labochamys segnis*) ^1,3^	Ara, Gal,^2^ Glu, Man, Rha, Xyl, UA	[49]

^1^ uronic acids were not detected in cell wall polysaccharides; ^2^ dominant sugar; ^3^ ribose was detected only in cell wall polysaccharides.

**Table 2:** Fatty acid (FA) profile of *C. reinhardtii*, *Spirulina* and *Chlorella*.

FA (DW)	*C. reinhardtii*		*Chlorella*		*Spirulina*	
	mg/g^-^	%	mg/g^-^	%	mg/g^-^	%
C16:0	16.7± 0.8	23.8 ± 0.2	11.0 ± 0.3	22.2 ± 0.1	25.9 ± 4.6	57.9 ± 0.4
C16:1 n-7	1.9 ± 0.1	2.7 ± 0.1	6.5 ± 0.1	13.0 ± 0.1	0.1 ± 0.0	0.1 ± 0.0
C16:4 n-3	3.9 ± 0.1	5.5 ± 0.3	0.0 ± 0.0	0.1 ± 0.0	0.0 ± 0.0	0.1 ± 0.0
C18:0	1.6 ± 0.2	2.3 ± 0.3	1.4 ± 0.0	2.8 ± 0.1	0.7± 0.0	1.5 ± 0.1
C18:1 n-9c	10.3 ± 1.1	14.7 ± 1.6	3.5 ± 0.1	7.0 ± 0.2	0.9 ± 0.2	2.0 ± 0.2
C18:2 n-6c	2.7 ± 0.2	3.8 ± 0.2	15.6 ± 0.3	31.4 ± 0.3	8.5 ± 1.6	19.0 ± 0.2
C18:3 n-6 (GLA)	2.9 ± 0.2	4.1 ± 0.3	0.0 ± 0.0	0.1 ± 0.0	8.7 ± 1.6	19.5 ± 0.2
C18:3 n-3	29.8 ± 1.9	42.4 ± 1.2	11.6 ± 0.2	23.4 ± 0.1	0.1 ± 0.0	0.1 ± 0.0
C20:4 n-6	0.6 ± 0.1	0.9 ± 0.0	0.0 ± 0.0	0.1 ± 0.0	8.7 ± 1.6	19.5 ± 0.2
ƩSFA	18.3	26.02	12.4	25.0	26.6	59.4
ƩUSFA	52.1	74.0	37.3	75.0	18.5	40.6
Ʃn-3 FA	33.7	47.9	11.6	23.4	0.1	0.2
Ʃn-6 FA	6.2	8.7	15.7	31.6	17.2	38.3
n-6/n-3	0.2	0.2	1.4	1.4	und	und

Abbreviations: DW: dry weight and und: undefined due to the very low amount. C16:0: palmitic acid; C16:1 n-7: palmitoleic acid; C16:4 n-3: hexadecatetraenoic acid; C18:0: stearic acid; C18:1 n-9c: oleic acid; C18:2 n-6c: linoleic acid (LA); C18:3 n-6: gamma-linolenic acid (GLA); C18:3 n-3: alpha-linolenic acid (ALA); C20:4 n-6: arachidonic acid; SFA: saturated fatty acid; USFA: unsaturated fatty acid. Reprinted from ref. [20].

**Table 3:** Overview of recombinant proteins produced in the chloroplast of *C. reinhardtii*. Adapted with permission from [114]. Copyright 2014 Springer.

Recombinant Therapeutic Protein	Yield	Ref.
VP1-CTB; protein VP1 from the foot and mouth disease virus (FMDV) fused to cholera toxin B (CTB)	3–4% total soluble protein (TSP)	[115]
HSV-lsc; large single chain (lsc) antibody directed against glycoprotein D protein from the herpes simplex virus (HSV)	Not reported	[116]
TRAIL; tumor necrosis factor-related apoptosis-inducing ligand	0.43–0.67% TSP	[117]
M-SAA; mammary-associated serum amyloid	3–5% TSP	[118]
CSFV-E2; classical swine fever virus (CSFV) structural protein E2	1.5–2% TSP	[119]
Human glutamic acid decarboxylase (hGAD65)	0.25–0.3% TSP	[120]
IBDV-VP2; infectious burial disease virus VP2 protein	4–0.8% total cell protein (TCP)	[121]
IHNV-G; infectious hematopoietic necrosis virus	<0.5% TCP	
IPNV-VP2; infectious pancreatic necrosis virus	<0.3% TCP	
VP2 protein	1–0.1% TCP	
IPNV-VP2 SBC; infectious pancreatic necrosis virus	1–0.2% TCP	
Quorum sensing-regulated gene (LecA) p57	<0.5 TCP	
PCV2; porcine circovirus type 2	0.9–0.2% TCP	
VP-2C	<0.5% TCP	
VP28	21–0.2% TCP	
HC-83K7C; heavy chain human monoclonal antibody against anthrax protective antigen 83 (PA83)	0.01% dry weight	[105]
LC-83K7C; light chain human monoclonal antibody against anthrax PA83		
CTB-D2; D2 fibronectin-binding domain of staphylococcus aureus fused to the cholera toxin B subunit	0.7% TSP	[23]
14FN3; domain 14 of human fibronectin	3–0.15% TSP	[122]
VEGF; human vascular endothelial growth factor	2–0.1% TSP	
HMGB1; high mobility group protein B1	2.5–1% TSP	
acrV2 and vapA2; antigens from the fish pathogen aeromonas salmonicida	0.8% and 0.3% TSPrespectively	[97]
Escherichia coli phytase gene (appA)	Not Detected	[123]
Pfs25 and Pfs28; surface proteins from plasmodium falciparum	0.5% and 0.2%TSP respectively	[124]
αCD22PE40; monomeric immunotoxin consisting of the single chain antibody that recognizes the CD22 surface protein from B- cells, fused to domains II and III of exotoxin A (PE40) from pseudomonas aeruginosa	0.3–0.4% TSP	[106]
αCD22HCH23PE40; dimeric version of αCD22PE40	0.2–0.3% TSP	
CtxB-Pfs25; plasmodium falciparum surface protein 25 fused to the β subunit of the cholera toxin from vibrio cholera	0.09% TSP	[24]
αCD22Gel; single-chain antibody targeting the CD22 receptor from B-cells, fused to the eukaryotic ribosome-inactivating protein, gelonin, from gelonium multiflorm	0.2–0.3% TSP	[112]
αCD22CH23Gel; dimeric version of αCD22Gel	0.1–0.2% TSP	

**Table 4:** Wild type *C. reinhardtii* (THN 6) dried biomass specifications (taken from the FDA application document [141] and Murbach et al. [142], and revised) and typical composition analysis results from two individual batches.

Physical Characteristics	Specification	Batch TAI-1215-01	Batch TAI-0316-01	Method
Appearance	Green powder	Green powder	^a^	Visual inspection
Water content	≤10%	5.6%	15.57%	AOAC Variable
**Composition**				
Protein	30–70%	36.0%	39.9%	AOAC 990.03
Fat	≤10%	2.0%	4.23%	AOAC 945.16
Fiber	1–25%	7.3%	3.6%	AOAC 991.43
Ash	≤5%	4.8%	1.87%	AOAC 942.05
Chlorophyll	≤25%	0.49%	^a^	[146]
**Heavy metals**				
Arsenic, cadmium, lead, Mercury	≤0.2 ppm	<0.1 ppm, 0.1 ppm, <0.1 ppm, <0.1 ppm	^a^	USP<233>, EPA 7471
**Microbiological tests**				
Total aerobic microbial	≤1000 CFU/g	ND	^a^	AOAC 990.12
Total yeast and mold	≤1000 CFU/g	190 CFU/g	^a^	BAM Ch. 18
Total coliforms	≤100 CFU/g	ND	^a^	AOAC 991.14
*E. coli*	Negative (absent/1 g)	Negative	^a^	AOAC 991.14
*Salmonella*	Negative (absent/25 g)	Negative	^a^	AOAC 030301
*Staphylococcus*	Negative (absent/1 g)	Negative	^a^	AOAC 2003.7

Abbreviations: BAM, US FDA Bacteriological Analytical Manual; CFU, colony forming units; EPA, US Environmental Protection Agency; ND, not detected; ppm, parts per million; USP, United States Pharmacopeia. ^a^ in accordance with the specifications [142].

## Data Availability

Not applicable.

## References

[1] Dyo Y.M., Purton S. (2018). The Algal Chloroplast as a Synthetic Biology Platform for Production of Therapeutic Proteins. Microbiology.

[2] Blanken W., Cuaresma M., Wijffels R.H., Janssen M. (2013). Cultivation of Microalgae on Artificial Light Comes at a Cost. Algal Res..

[3] Chisti Y. (2007). Biodiesel from Microalgae. Biotechnol. Adv..

[4] Kay R.A., Barton L.L. (1991). Microalgae as Food and Supplement. Crit. Rev. Food Sci. Nutr..

[5] Potijun S., Yaisamlee C., Sirikhachornkit A. (2021). Pigment Production under Cold Stress in the Green Microalga *Chlamydomonas* reinhardtii. Agriculture.

[6] Kiran B.R., Venkata Mohan S. (2021). Microalgal Cell Biofactory—Therapeutic, Nutraceutical and Functional Food Applications. Plants.

[7] Gifuni I., Pollio A., Safi C., Marzocchella A., Olivieri G. (2019). Current Bottlenecks and Challenges of the Microalgal Biorefinery. Trends Biotechnol..

[8] Harris E.H. (2001). Chlamydomonas as a model organism. Annu. Rev. Plant Physiol. Plant Mol. Biol..

[9] Harris E.H., Stern D.B., Witman G.B. (1989). Organellar and Metabolic Processes. The Chlamydomonas Sourcebook.

[10] Sasso S., Stibor H., Mittag M., Grossman A.R. (2018). The natural history of model organisms: From molecular manipulation of domesticated *Chlamydomonas reinhardtii* to survival in nature. Elife.

[11] Harris E.H., Harris E.H., Stern D.B., Witman G.B. (2009). Introduction to Chlamydomonas and Its Laboratory Use. The Chlamydomonas Sourcebook.

[12] Pröschold T., Harris E.H., Coleman A.W. (2005). Portrait of a species: *Chlamydomonas reinhardtii*. Genetics.

[13] Merchant S.S., Prochnik S.E., Vallon O., Harris E.H., Karpowicz S.J., Witman  G.B., Terry A., Salamov A., Fritz-Laylin L.K., Maréchal-Drouard L. (2007). The Chlamydomonas genome reveals the evolution of key animal and plant functions. Science.

[14] Rochaix J.D. (1995). *Chlamydomonas reinhardtii* as the photosynthetic yeast. Annul. Rev. Genet..

[15] Blaby I.K., Blaby-Haas C.E., Tourasse N., Hom E.F.Y., Lopez D., Aksoy M., Grossman A., Umen J., Dutcher S., Porter M. (2014). The Chlamydomonas genome project: A decade on. Trends Plant Sci..

[16] Goodstein D.M., Shu S., Howson R., Neupane R., Hayes R.D., Fazo J., Mitros T., Dirks W., Hellsten U., Putnam N. (2012). Phytozome: A comparative platform for green plant genomics. Nucleic Acids Res..

[17] Bule M.H., Ahmed I., Maqbool F., Bilal M., Iqbal H.M. (2018). Microalgae as a source of high- value bioactive compounds. Front. Biosci..

[18] Jayshree A., Jayashree S., Thangaraju N. (2016). *Chlorella vulgaris* and *Chlamydomonas reinhardtii*: Effective antioxidant, antibacterial and anticancer mediators. Indian J. Pharm. Sci..

[19] Annamalai J., Nallamuthu T. (2014). Antioxidant potential phytochemicals from methanol extract of *Chlorella vulgaris* and *Chlamydomonas reinhardtii*. J. Algal Biomass Util.

[20] Darwish R., Gedi M.A., Akepach P., Assaye H., Zaky A.S., Gray D.A. (2020). *Chlamydomonas reinhardtii* Is a Potential Food Supplement with the Capacity to Outperform Chlorella and Spirulina. Appl. Sci..

[21] Scranton M.A., Ostrand J.T., Fields F.J., Mayfield S.P. (2015). Chlamydomonas as a model for biofuels and bio-products production. Plant J..

[22] Zhang Z., Tan Y., Wang W., Bai W., Fan J., Huang J., Wan M., Li Y. (2018). Efficient heterotrophic cultivation of *Chlamydomonas Reinhardtii*. J. Appl. Phycol..

[23] Dreesen I.A., Charpin-El Hamri G., Fussenegger M. (2010). Heat-stable oral alga-based vaccine protects mice from *Staphylococcus aureus* infection. J. Biotechnol..

[24] Gregory J.A., Topol A.B., Doerner D.Z., Mayfield S.P. (2013). Alga-produced cholera toxin- Pfs25 fusion proteins as oral vaccines. Appl. Environ. Microbiol..

[25] Torres-Tiji Y., Fields F.J., Mayfield S.P. (2020). Microalgae as a future food source. Biotechnol. Adv..

[26] Caporgno M.P., Mathys A. (2018). Trends in microalgae incorporation into innovative food products with potential health benefits. Front. Nut..

[27] Lordan S., Ross R.P., Stanton C. (2011). Marine bioactives as functional food ingredients: Potential to reduce the incidence of chronic diseases. Mar. Drugs.

[28] Nuño K., Villarruel-López A., Puebla-Pérez A.M., Romero-Velarde E., Puebla- Mora A.G., Ascencio F. (2013). Effects of the marine microalgae *Isochrysis galbana* and *Nannochloropsis oculata* in diabetic rats. J. Funct. Foods.

[29] Deng R., Chow T.J. (2010). Hypolipidemic, antioxidant, and antiinflammatory activities of microalgae spirulina. Cardiovasc. Ther..

[30] Fallah A.A., Sarmast E., Dehkordi S.H., Engardeh J., Mahmoodnia L., Khaledifar A., Jafari T. (2018). Effect of Chlorella supplementation on cardiovascular risk factors: A meta-analysis of randomized controlled trials. Clin. Nutr..

[31] Fields F.J., Lejzerowicz F., Schroeder D., Ngoi S.M., Tran M., McDonald D., Jiang L., Chang J.T., Knight R., Mayfield S. (2020). Effects of the microalgae Chlamydomonas on gastrointestinal health. J. Funct. Foods.

[32] Bhowmick S., Mazumdar A., Moulick A., Adam V. (2020). Algal metabolites: An inevitable substitute for antibiotics. Biotechnol. Adv..

[33] Vishwakarma J., Vavilala S.L. (2019). Evaluating the antibacterial and antibiofilm potential of sulphated polysaccharides extracted from green algae *Chlamydomonas reinhardtii*. J. Appl. Microbiol..

[34] Kamble P., Cheriyamundath S., Lopus M., Sirisha V.L. (2018). Chemical characteristics, antioxidant and anticancer potential of sulfated polysaccharides *from Chlamydomonas reinhardtii*. J. Appl. Phycol..

[35] Mitchell S.F., Trainor F.R., Rich P.H., Goulden C.E. (1992). Growth of Daphnia Magna in the Laboratory in Relation to the Nutritional State of Its Food Species, *Chlamydomonas Reinhardtii*. J. Plankton Res..

[36] Davies J.P., Grossman A.R. (1998). The use of Chlamydomonas (Chlorophyta: Volvocales) as a model algal system for genome studies and the elucidation of photosynthetic processes. J. Phycol..

[37] Vukavic T. (1983). Intestinal Absorption of IgA in the Newborn. J. Pediatr. Gastroenterol. Nutr..

[38] Yang B., Liu J., Jiang Y., Chen F. (2016). Chlorella Species as Hosts for Genetic Engineering and Expression of Heterologous Proteins: Progress, Challenge and Perspective. Biotechnol. J..

[39] Lönnerdal B. (2014). Infant Formula and Infant Nutrition: Bioactive Proteins of Human Milk and Implications for Composition of Infant Formulas. Am. J. Clin. Nutr..

[40] Guedes A.C., Amaro H.M., Malcata F.X. (2011). Microalgae as Sources of Carotenoids. Mar. Drugs.

[41] Hou Q., Qiu S., Liu Q., Tian J., Hu Z., Ni J. (2013). Selenoprotein-Transgenic Chlamydomonas Reinhardtii. Nutrition.

[42] Burgess J.G. (2012). New and emerging analytical techniques for marine biotechnology. Curr. Opin. Biotechnol..

[43] Bafana A. (2013). Characterization and optimization of production of exopolysaccharide from *Chlamydomonas reinhardtii*. Carbohydr. Polym..

[44] Díaz-Montes E. (2022). Polysaccharides: Sources, Characteristics, Properties, and Their Application in Biodegradable Films. Polysaccharides.

[45] Sousa A.M.M., Rocha C.M.R., Gonçalves M.P., Phillips G.O., Williams P.A. (2021). Agar. Handbook of Hydrocolloids.

[46] Morais M.G., Santos T.D., Moraes L., Vaz B.S., Morais E.G., Costa J.A.V. (2022). Exopolysaccharides from microalgae: Production in a biorefinery framework and potential applications. Bioresour. Technol. Rep..

[47] Zhang J., Liu L., Ren Y., Chen F. (2019). Characterization of exopolysaccharides produced by microalgae with antitumor activity on human colon cancer cells. Int. J. Biol. Macromol.

[48] Borowitzka M.A., Beardall J., Raven J.A. (2016). The Physiology of Microalgae, Developments in Applied Phycology 6.

[49] Barclay R.W., Lewin R.A. (1985). Microalgal polysaccharide production for the conditioning of agricultural soils. Plant Soil.

[50] Choi S.P., Nguyen M.T., Sim S.J. (2010). Enzymatic pretreatment of *Chlamydomonas reinhardtii* biomass for ethanol production. Bioresour. Technol..

[51] Ball S.G., Dirick L., Decq A., Martiat J.C., Matagne R. (1990). Physiology of starch storage in the monocellular alga *Chlamydomonas reinhardtii*. Plant Sci.

[52] Melis A. (2007). Photosynthetic H_2_ metabolism in *Chlamydomonas reinhardtii* (unicellular green algae). Planta.

[53] Raposo M.F.J., de Morais A.M.B., de Morais R.M.S.C. (2015). Marine polysaccharides from algae with potential biomedical applications. Mar. Drugs.

[54] Wijesekara I., Pangestuti R., Kim S.K. (2011). Biological activities and potential health benefits of sulphated polysaccharides derived from marine algae. Carbohyd. Polym.

[55] Costa L.S., Fidelis G.P., Cordeiro S.L., Oliveira R.M., Sabry D.A., Câmara R.B.G., Nobre L.T.D.B., Costa M.S.S.P., Almeida-Lima J., Farias E.H.C. (2010). Biological activities of sulfated polysaccharides from tropical seaweeds. Biomed Pharm..

[56] Morais A.M.M.B., Alves A., Kumla D., Morais R.M.S.C., Oliveira J.M., Radhouani H., Reis R.L. (2021). Pharmaceutical and Biomedical Potential of Sulphated Polysaccharides from Algae. Polysaccharides of Microbial Origin.

[57] Camara R.B., Day L.S., Fidelis G.P., Nobre L.T., Dantas-Santos N., Cordiro S.L., Costa M.S., Alves L.G., Rocha H.A. (2011). Heterofucans from the brown seaweed *Canistrocarpus cervicornis* with anticoagulant and antioxidant activities. Mar. Drugs.

[58] Rodrigues J.A.G., Neto E.M., Teixeira L.A.C., Maula P.R.C., Mourao P.A.S., Benevides N.M.B. (2013). Structural features and inactivation of coagulation proteases of a sulfated polysaccharidic fraction from Caulerpa cupressoides varlycopodium (Caulerpaceae, Chlorophyta.). Acta Sci. Technol..

[59] Faggio C., Pagano M., Dottorem A., Genovese G., Morabito M. (2016). Evaluation of anticoagulant activity of two algal polysaccharides. Nat. Prod. Res.

[60] Necas J., Bartosikova L. (2013). Carrageenan: A review. Vet. Med..

[61] Ahmadi A., Moghadamtousi S.Z., Abubakar S., Zandi K. (2015). Antiviral potential of algae polysaccharides isolated from marine sources: A Review. Biomed Res. Int..

[62] Hu T.D., Liu D., Chen Y., Wu J., Wang S. (2010). Antioxidant activity of sulfated polysaccharide fractions extracted from *Undaria pinnitafida in vitro*. Int. J. Biol. Macromol..

[63] Costa L.S., Fidelis G.P., Telles C.B.S., Dantas-Santos N., Camara R.B.G.S., Cordeiro L., Pereira Costa M.S.S., Almeida-Lima J., Melo-Silveira R.F., Oliveira R.M. (2011). Antioxidant and anti-proliferative activities of heterofucans from the seaweed *Sargassum filipendula*. Mar. Drugs.

[64] Souza B.W.S., Cerqueira M.A., Bourbon A.I., Pinheiro A.C., Martins J.T., Teixeira J.A., Coimbra M.A., Vicente A.A. (2012). Chemical characterization and antioxidant activity of sulfated polysaccharide from the red seaweed *Gracilaria birdiae*. Food Hydrocoll..

[65] Wang J., Hu S., Nie S., Yu Q., Xie M. (2016). Reviews on mechanisms of In vitro antioxidant activity of polysaccharides. Oxid. Med. Cell. Longev..

[66] Luo M., Shao B., Nie W., Wei X.W., Li Y.L., Wang B.L., He Z.Y., Liang X., Ye T.H., Wei Y.Q. (2015). Antitumor and adjuvant activity of λ-carrageenan by stimulating immune response in cancer immunotherapy. Sci.Rep..

[67] Lowenthal R.M., Fitton J.H. (2015). Are seaweed-derived fucoidans possible future anti-cancer agents?. J. Appl. Phycol..

[68] Shao P., Chen X., Sun P. (2013). In vitro antioxidant and antitumor activities of different sulfated polysaccharides isolated from three algae. Int. J. Biol. Macromol..

[69] Zaporozhets T.S., Ermakova S.V., Zvyagintseva T.N., Besednova N.N. (2014). Antitumor effects of sulphated polysaccharides produced from marine algae. Biol. Bull. Rev.

[70] Rodrigues J.A.G., de Queiroz I.N.L., Gomes Quinderé A.L., Vairo B.C., de Souza Mourão P.A., Benevides N.M.B. (2011). An antithrombin-dependent sulfated polysaccharide isolated from the green alga Caulerpa cupressoides has in vivo anti- and prothrombotic effects. Ciência Rural.

[71] Amorim R.D., Rodrigues J.A., Holanda M.L., Quinderé A.L., Paula R.C., Melo V.M., Benevides N.M. (2012). Antimicrobial effect of a crude sulfated polysaccharide from the red seaweed *Gracilaria ornate*. Braz. Arch. Biol. Technol..

[72] Maeda H., Hosokawa M., Sashima T., Miyashita K. (2007). Dietary combination of fucoxanthin and fish oil attenuates the weight gain of white adipose tissue and decreases blood glucose in obese/diabetic KK-Ay Mice. J. Agric. Food Chem..

[73] Tsukui T., Konno K., Hosokawa M., Maeda H., Sashima T., Miyashita K. (2007). Fucoxanthin and fucoxanthinol enhance the amount of docosahexaenoic acid in the liver of KKAy obese/diabetic mice. J. Agric. Food Chem..

[74] Kong C.S., Kim J., Ahn B.N., Vo T.S., Yoon N.Y., Kim S.K. (2001). 1-(3, 5-Dihydroxyphenoxy)-7-(2, 4, 6- trihydroxyphenoxy)-2, 4, 9-trihydroxydibenzo-1, 4-dioxin inhibits adipocyte differentiation of 3T3-L1 fibroblasts. Mar. Biotechnol..

[75] Marques C.T., de Azevedo T.C.G., Nascimento M.S., Medeiros V.P., Alves L.G., Benevides N.M.B., Rocha H.A.O., Leite E.L. (2012). Sulfated fucans extracted from algae *Padina gymnospora* have Anti-inflammatory effect. Rev. Bras. Farmacogn..

[76] Coura C.O., Souza R.B., Rodrigues J.A., Vanderlei E.D., de Araújo I.W., Ribeiro N.A., Frota A.F., Ribeiro K.A., Chaves H.V., Pereira K.M. (2015). Mechanisms involved in the anti-inflammatory action of a polysulfated fraction from Gracilaria cornea in rats. PLoS ONE.

[77] Carneiro J.G., Rodrigues J.A.G., de Sousa Oliveira Vanderlei E., Basto Souza R., Quinderé A.L.G., Coura C.O., de Araújo I.W.F., Chaves H.V., Bezerra M.M., Benevides N.M.B. (2014). Peripheral Antinociception and Anti-Inflammatory Effects of Sulphated Polysaccharides from the Alga *Caulerpa Mexicana*. Basic Clin. Pharm. Toxicol..

[78] He F., Yang Y., Yang G., Yu L.J. (2010). Studies on antibacterial activity and antibacterial mechanism of a novel polysaccharide from *Streptomyces virginia* H03. Food Control.

[79] Choudhary S., Save S.N., Vavilala S.L. (2018). Unravelling the inhibitory activity of *Chlamydomonas reinhardtii* sulfated polysaccharides against α-Synuclein fibrillation. Sci. Rep..

[80] Somerville C., Browse J., Jaworski J., Ohlrogge J., Buchanan B.B., Gruissem W., Jones R.L. (2000). Lipids. Biochemistry and Molecular Biology of Plants.

[81] Lu C., Napier J.A., Clemente T.E., Cahoon E.B. (2011). New frontiers in oilseed biotechnology: Meeting the global demand for vegetable oils for food, feed, biofuel, and industrial applications. Curr. Opin. Biotechnol..

[82] Breuer G., Lamers P.P., Martens D.E., Draaisma R.B., Wijffels R.H. (2012). The impact of nitrogen starvation on the dynamics of triacylglycerol accumulation in nine microalgae strains. Bioresour. Technol..

[83] Affudeen C.L.W., Loh S.H., Aziz A., Takahashi K., Efendy A.W.M., Cha T.S. (2021). Double-high in palmitic and oleic acids accumulation in a non-model green microalga, *Messastrum gracile* SE-MC4 under nitrate-repletion and -starvation cultivations. Sci. Rep..

[84] Riediger N.D., Othman R.A., Suh M., Moghadasian M.H. (2009). A systemic review of the roles of n-3 fatty acids in health and disease. J. Am. Diet. Assoc..

[85] Khozin-Goldberg I., Leu S., Boussiba S. (2016). Microalgae as a source for VLC-PUFA production. Lipids Plant Algae Dev..

[86] Calder P.C. (2013). Omega-3 polyunsaturated fatty acids and inflammatory processes: Nutrition or pharmacology?. Br. J. Clin. Pharmacol..

[87] Calder P.C. (2009). Polyunsaturated fatty acids and inflammatory processes: New twists in an old tale. Biochimie.

[88] Ferreri C., Chatgilialoglu C. (2015). Membrane Lipidomics for Personalized Health.

[89] Shahidi F., Ambigaipalan P. (2018). Omega-3 polyunsaturated fatty acids and their health benefits. Annu. Rev. Food Sci. Technol..

[90] Solovchenko A.E. (2012). Physiological role of neutral lipid accumulation in eukaryotic microalgae under stresses. Russ. J. Plant Physiol..

[91] Mulgund A. (2022). Increasing Lipid Accumulation in Microalgae through Environmental Manipulation, Metabolic and Genetic Engineering: A Review in the Energy NEXUS framework. Energy Nexus.

[92] Mata T.M., Martins A.A., Caetano N.S. (2010). Microalgae for biodiesel production and other applications: A review. Renew. Sustain. Energy Rev..

[93] Li-Beisson Y., Beisson F., Riekhof W. (2015). Metabolism of acyl-lipids in *Chlamydomonas reinhardtii*. Plant J..

[94] Figueroa-Torres G.M., Pittman J.K., Theodoropoulos C. (2021). Optimisation of Microalgal Cultivation via Nutrient-Enhanced Strategies: The Biorefinery Paradigm. Biotechnol. Biofuels.

[95] Ferreri C., Masi A., Sansone A., Giacometti G., Larocca A.V., Menounou G., Scanferlato R., Tortorella S., Rota D., Conti M. (2017). Fatty acids in membranes as homeostatic, metabolic and nutritional biomarkers: Recent advancements in analytics and diagnostics. Diagnostics.

[96] Day A., Goldschmidt-Clermont M. (2011). The chloroplast transformation toolbox: Selectable markers and marker removal. Plant Biotechnol. J..

[97] Michelet L., Lefebvre-Legendre L., Burr S.E., Rochaix J.D., Goldschmidt-Clermont M. (2011). Enhanced chloroplast transgene expression in a nuclear mutant of Chlamydomonas. Plant Biotechnol. J..

[98] Rochaix J.D., Beatty J.T., Gest H., Allen J.F. (2005). The three genomes of Chlamydomonas. Discoveries in Photosynthesis, Govindjee.

[99] Borowiak D., Krzywonos M. (2022). Bioenergy, biofuels, lipids and pigments—Research trends in the use of microalgae grown in photobioreactors. Energies.

[100] De Luca M., Pappalardo I., Limongi A.R., Viviano E., Radice R.P., Todisco S., Martelli G., Infantino V., Vassallo A. (2021). Lipids from microalgae for cosmetic applications. Cosmetics.

[101] Khan M.I., Shin J.H., Kim J.D. (2018). The promising future of microalgae: Current status, challenges, and optimization of a sustainable and renewable industry for biofuels, feed, and other products. Microb. Cell Factories.

[102] Zhang M.P., Wang M., Wang C. (2021). Nuclear transformation of *Chlamydomonas reinhardtii*: A review. Biochimie.

[103] Cutolo E.A., Mandalà G., Dall’Osto L., Bassi R. (2022). Harnessing the Algal Chloroplast for Heterologous Protein Production. Microorganisms.

[104] Braun-Galleani S., Baganz F., Purton S. (2015). Improving recombinant protein production in the Chlamydomonas reinhardtii chloroplast using vivid Verde Fluorescent Protein as a reporter. Biotechnol. J..

[105] Tran M., Zhou B., Pettersson P.L., Gonzalez M.J., Mayfield S.P. (2009). Synthesis and assembly of a full-length human monoclonal antibody in algal chloroplasts. Biotechnol. Bioeng..

[106] Tran M., Van C., Barrera D.J., Pettersson P.L., Peinado C.D., Bui J., Mayfield S.P. (2013). Production of unique immunotoxin cancer therapeutics in algal chloroplasts. Proc. Natl. Acad. Sci. USA.

[107] Chávez M.N., Schenck T.L., Hopfner U., Centeno-Cerdas C., Somlai-Schweiger I., Schwarz C., Machens H.G., Heikenwalder M., Bono M.R., Allende M.L. (2016). Towards autotrophic tissue engineering: Photosynthetic gene therapy for regeneration. Biomaterials.

[108] Schroda M. (2004). The Chlamydomonas genome reveals its secrets: Chaperone genes and the potential roles of their gene products in the chloroplast. Photosynth. Res..

[109] Breiman A., Fawcett T.W., Ghirardi M.L., Mattoo A.K. (1992). Plant organelles contain distinct peptidylprolyl cis, trans-isomerases. J. Biol. Chem..

[110] Ramana K.V., Xavier J.R., Sharma R.K. (2017). Recent trends in pharmaceutical biotechnology. Pharm. Biotechnol. Curr. Res..

[111] Yan N., Fan C., Chen Y., Hu Z. (2016). The potential for microalgae as bioreactors to produce pharmaceuticals. Int. J. Mol. Sci..

[112] Pacheco S.E.C., Hankamer B., Oey M. (2018). Optimising light conditions increases recombinant protein production in *Chlamydomonas reinhardtii* chloroplasts. Algal Res..

[113] Stoffels L., Finlan A., Mannall G., Purton S., Parker B. (2019). Downstream processing of Chlamydomonas reinhardtii TN72 for recombinant protein recovery. Front. Bioeng. Biotechnol..

[114] Almaraz-Delgado A.L., Flores-Uribe J., Pérez-España V.H., Salgado-Manjarrez E., Badillo-Corona J.A. (2014). Production of therapeutic proteins in the chloroplast of *Chlamydomonas reinhardtii*. AMB Express.

[115] Sun M., Qian K., Su N., Chang H., Liu J., Shen G. (2003). Foot-and-mouth disease virus VP1 protein fused with cholera toxin B subunit expressed in *Chlamydomonas reinhardtii* chloroplast. Biotechnol. Lett..

[116] Mayfield S.P., Franklin S.E., Lerner R.A. (2003). Expression and assembly of a fully active antibody in algae. Proc. Natl. Acad. Sci. USA.

[117] Yang Z., Chen F., Li D., Zhang Z., Liu Y., Zheng D., Liu Yanxin L., Dexian Z., Yong W., Shen G. (2006). Expression of human soluble TRAIL in *Chlamydomonas reinhardtii* chloroplast. Chin. Sci. Bull..

[118] Manuell A.L., Beligni M.V., Elder J.H., Siefker D.T., Tran M., Weber A., McDonal T.L., Mayfield S.P. (2007). Robust expression of a bioactive mammalian protein in Chlamydomonas chloroplast. Plant Biotechnol. J..

[119] He D.M., Qian K.X., Shen G.F., Zhang Z.F., Li Y.N., Su Z.L., Shao H.B. (2007). Recombination and expression of classical swine fever virus (CSFV) structural protein E2 gene in *Chlamydomonas reinhardtii* chroloplasts. Colloids Surf. B Biointerfaces.

[120] Wang X., Brandsma M., Tremblay R., Maxwell D., Jevnikar A.M., Huner N., Ma S. (2008). A novel expression platform for the production of diabetes-associated autoantigen human glutamic acid decarboxylase (hGAD65). BMC Biotechnol..

[121] Surzycki R., Greenham K., Kitayama K., Dibal F., Wagner R., Rochaix J.D., Ajam T., Surzycki S. (2009). Factors effecting expression of vaccines in microalgae. Biologicals.

[122] Rasala B.A., Muto M., Lee P.A., Jager M., Cardoso R.M., Behnke C.A., Kirk C.A., Hokanson R., Crea M., Mendez S.P. (2010). Production of therapeutic proteins in algae, analysis of expression of seven human proteins in the chloroplast of *Chlamydomonas reinhardtii*. Plant Biotechnol. J..

[123] Yoon S.M., Kim S.Y., Li K.F., Yoon B.H., Choe S., Kuo M.M.C. (2011). Transgenic microalgae expressing *Escherichia coli* AppA phytase as feed additive to reduce phytate excretion in the manure of young broiler chicks. Appl. Microbiol. Biot..

[124] Gregory J.A., Li F., Tomosada L.M., Cox C.J., Topol A.B., Vinetz J.M., Mayfield S. (2012). Algae-produced Pfs25 elicits antibodies that inhibit malaria transmission. PLoS ONE.

[125] Tran M., Henry R.E., Siefker D., Van C., Newkirk G., Kim J., Bui J., Mayfield S.P.M., Tran R.E. (2013). Production of anti-cancer immunotoxins in algae: Ribosome inactivating proteins as fusion partners. Biotechnol. Bioeng..

[126] Rasala B.A., Mayfield S.P. (2011). The microalga *Chlamydomonas reinhardtii* as a platform for the production of human protein therapeutics. Bioeng. Bugs.

[127] Pang X., Tong Y., Xue W., Yang Y.F., Chen X., Liu J., Chen D. (2019). Expression and characterization of recombinant human lactoferrin in edible alga *Chlamydomonas reinhardtii*. Biosci. Biotechnol. Biochem..

[128] Grossman A.R., Lohr M., Im C.S. (2004). *Chlamydomonas reinhardtii* in the landscape of pigments. Annu. Rev. Genet..

[129] Koyande A.K., Chew K.W., Rambabu K., Tao Y., Chu D.T., Show P.L. (2019). Microalgae: A potential alternative to health supplementation for humans. Food Sci. Hum. Wellness.

[130] Gille A., Trautmann A., Posten C., Briviba K. (2016). Bioaccessibility of carotenoids from *Chlorella vulgaris* and *Chlamydomonas reinhardtii*. Int. J. Food Sci. Nutr.

[131] Rathod J.P., Vira C., Lali A.M., Prakash G. (2010). Metabolic engineering of *Chlamydomonas reinhardtii* for enhanced β-carotene and lutein production. Appl. Biochem. Biotechnol..

[132] Song I., Kim J., Baek K., Choi Y., Shin B., Jin E. (2020). The generation of metabolic changes for the production of high-purity zeaxanthin mediated by CRISPR-Cas9 in *Chlamydomonas reinhardtii*. Microb. Cell Factories.

[133] Cordero B.F., Couso I., León R., Rodríguez H., Vargas M.A. (2011). Enhancement of carotenoids biosynthesis in *Chlamydomonas reinhardtii* by nuclear transformation using a phytoene synthase gene isolated from *Chlorella zofingiensis*. Appl. Microbiol. Biotechnol..

[134] Zhao X., Ma R., Liu X., Ho S.H., Xie Y., Chen J. (2019). Strategies related to light quality and temperature to improve lutein production of marine microalga Chlamydomonas sp.. Bioprocess Biosyst. Eng..

[135] Tran Q.G., Cho K., Kim U., Yun J.H., Cho D.H., Heo J., Park S.B., Kim J.W., Lee Y.J., Ramanan R. (2019). Enhancement of β-carotene production by regulating the autophagy- carotenoid biosynthesis seesaw in *Chlamydomonas reinhardtii*. Bioresour. Technol..

[136] Scott J.D., Chalker-Scott L., Foreman A.E., D’Angelo M. (1999). *Daphnia Pulex* Fed UVB- Irradiated *Chlamydomonas Reinhardtii* Show Decreased Survival and Fecundity. Photochem. Photobioliol..

[137] Gophen M. (1977). Feeding of *Daphnia* on *Chlamydomonas* and *Chlorobium*. Nature.

[138] Taub F.B., Dollar A.M. (1968). The nutritional inadequacy of *Chlorella* and *Chlamydomonas* as food for *Daphnia Pulex*. Limnol. Oceanogr..

[139] Weers P.M.M., Gulati R.D. (1997). Gulati, Growth and Reproduction of Daphnia Galeata in Response to Changes in Fatty Acids, Phosphorus, and Nitrogen in Chlamydomonas Reinhardtii. Limnol. Oceanogr..

[140] DeMott W.R. (1982). Feeding Selectivities and Relative Ingestion Rates of Daphnia and Bosmina. Limnol. Oceanogr..

[141] Triton Algae Innovations Inc. Notice to US Food and Drug Administration of the Conclusion that the Intended Use of Chlamydomonas reinhardtii (THN 6) Dried Biomass Powder is Generally Recognized as Safe. https://www.fda.gov/media/128921/download.

[142] Murbach T.S., Glávits R., Endres J.R., Hirka G., Vértesi A., Béres E., Szakonyiné I.P. (2018). Toxicological Evaluation of *Chlamydomonas Reinhardtii*, a Green Algae. Int. J. Toxicol..

[143] Becker E.W. (2007). Micro-Algae as a Source of Protein. Biotechnol. Adv..

[144] Boyle N.R., Morgan J.A. (2009). Flux Balance Analysis of Primary Metabolism in *Chlamydomonas Reinhardtii*. BMC Syst. Biol..

[145] Griffiths M.J., Harrison S.T.L. (2008). Lipid Productivity as a Key Characteristic for Choosing Algal Species for Biodiesel Production. J. Appl. Phycol..

[146] Knap A.H., Michaels A., Close A.R., Ducklow H., Dickson A.G. (1994). Protocols for the Joint Global Ocean Flux Study (JGOFS) Core Measurements. Intergovernmental Oceanographic Commission.

[147] Baosheng G.E., Xiangfa W., Fang H., Jie L. (2019). Method for Overproducing Hemoglobin in Algae and Compositions Therefrom. Chinese Patent.

[148] Wang X., Innovations T.A. Green Algae as a Platform for Protein Production: Food, Feed, and Nutritional Supplements; Montreal, QC, Canada, 2017. https://www.bio.org/sites/default/files/legacy/bioorg/docs/0830AM-Xun%20Wang.pdf.

[149] Tran M., Deaton J., Adams B., Mayfield M., Longo A., Gonzalez O., Hansen J., Wang X., Schroeder D. (2021). Compositions and Methods for Incorporating Heme from Algae in Edible Products. U.S. Patent.

[150] Tran M., Deaton J., Adams B., Mayfield M., Longo A., Gonzalez O., Hansen J., Wang X., Schroeder D. (2019). Method of Purification of Recombinant Osteopontin from Microalgae. U.S. Patent.

[151] Tran M., Deaton J., Adams B., Mayfield M., Longo A., Gonzalez O., Hansen J., Wang X., Schroeder D. (2021). Methods for Overproducing Protoporphyrin Ix in Algae and Compositions Therefrom. U.S. Patent.

[152] Diego S., Adams B., Diego S., Deaton J., Diego S., Hansen J., Diego S., Longo A., Diego S., Mayfield M. (2020). Production of Iron—Complexed Proteins. U.S. Patent.

[153] Peña D.A., Gasser B., Zanghellini J., Steiger M.G., Mattanovich D. (2018). Metabolic Engineering of Pichia Pastoris. Metab. Eng..

[154] Fraser R.Z., Shitut M., Agrawal P., Mendes O., Klapholz S. (2018). Safety Evaluation of Soy Leghemoglobin Protein Preparation Derived From Pichia Pastoris, Intended for Use as a Flavor Catalyst in Plant-Based Meat. Int. J. Toxicol..

[155] Sproles A.E., Fields F.J., Smalley T.N., Le C.H., Badary A., Mayfield S.P. (2021). Recent advancements in the genetic engineering of microalgae. Algal Res..

[156] Crozet P., Navarro F.J., Willmund F., Mehrshahi P., Bakowski K., Lauersen K.J., Pérez-Pérez M.-E., Auroy P., Gorchs Rovira A., Sauret-Gueto S. (2018). Birth of a photosynthetic chassis: A MoClo toolkit enabling synthetic biology in the microalga *Chlamydomonas reinhardtii*. ACS Synth. Biol..

[157] Weiner I., Atar S., Schweitzer S., Eilenberg H., Feldman Y., Avitan M., Blau M., Danon A., Tuller T., Yacoby I. (2018). Enhancing heterologous expression in *Chlamydomonas reinhardtii* by transcript sequence optimization. Plant J..

[158] Ramos-Martinez E.M., Fimognari L., Sakuragi Y. (2017). High-yield secretion of recombinant proteins from the microalga *Chlamydomonas reinhardtii*. Plant Biotechnol. J..

[159] Rasala B.A., Mayfield S.P. (2015). Photosynthetic biomanufacturing in green algae; production of recombinant proteins for industrial, nutritional, and medical uses. Photosynth. Res..

[160] Schroda M., Blöcker D., Beck C.F. (2000). The HSP70A promoter as a tool for the improved expression of transgenes in *Chlamydomonas*. Plant J..

[161] Perozeni F., Stella G.R., Ballottari M. (2018). LHCSR expression under HSP70/RBCS2 promoter as a strategy to increase productivity in microalgae. Int. J. Mol. Sci..

[162] Xue B., Dong C.M., Hu H.H., Dong B., Fan Z.C. (2020). *Chlamydomonas reinhardtii*-expressed multimer of ToAMP4 inhibits the growth of bacteria of both Gram-positive and Gram- negative. Process Biochem..

[163] Kwon K.C., Lamb A., Fox D., Jegathese S.J.P. (2019). An evaluation of microalgae as a recombinant protein oral delivery platform for fish using green fluorescent protein (GFP). Fish Shellfish Immunol..

[164] Shamriz S., Ofoghi H. (2019). Expression of recombinant PfCelTOS antigen in the chloroplast of *Chlamydomonas reinhardtii* and its potential use in detection of malaria. Mol. Biotechnol..

[165] Rosales-Mendoza S., Solís-Andrade K.I., Márquez-Escobar V.A., González-Ortega O., Bañuelos-Hernandez B. (2020). Current advances in the algae-made biopharmaceuticals field. Expert Opin. Biol. Ther..

[166] Griesbeck C., Kobl I., Heitzer M. (2006). Chlamydomonas reinhardtii. Mol. Biotechnol..

[167] Perozeni F., Cazzaniga S., Baier T., Zanoni F., Zoccatelli G., Lauersen K.J., Wobbe L., Ballottari M. (2020). Turning a green alga red: Engineering astaxanthin biosynthesis by intragenic pseudogene revival in *Chlamydomonas reinhardtii*. Plant Biotechnol. J..

[168] Gaj T., Gersbach C.A., Barbas C.F. (2013). ZFN, TALEN, and CRISPR/Cas-based methods for genome engineering. Trends Biotechnol..

[169] Antonacci A., Scognamiglio V. (2020). Biotechnological advances in the design of algae-based biosensors. Trends Biotechnol..

[170] Ghribi M., Nouemssi S.B., Meddeb-Mouelhi F., Desgagné-Penix I. (2020). Genome editing by CRISPR-Cas: A game change in the genetic manipulation of Chlamydomonas. Life.

[171] Shin S.E., Lim J.M., Koh H.G., Kim E.K., Kang N.K., Jeon S., Kwon S., Shin W.S., Lee B., Hwangbo K. (2016). CRISPR/Cas9-induced knockout and knock-in mutations in *Chlamydomonas reinhardtii*. Sci. Rep..

[172] Baek K., Yu J., Jeong J., Sim S.J., Bae S., Jin E. (2018). Photoautotrophic production of macular pigment in a Chlamydomonas reinhardtii strain generated by using DNA-free CRISPR-Cas9 RNP-mediated mutagenesis. Biotechnol. Bioeng..

[173] Rasala B.A., Chao S.S., Pier M., Barrera D.J., Mayfield S.P. (2014). Enhanced Genetic Tools for Engineering Multigene Traits into Green Algae. PLoS ONE.

[174] Scaife M.A., Nguyen G.T.D.T., Rico J., Lambert D., Helliwell K.E., Smith A.G. (2015). Establishing *Chlamydomonas Reinhardtii* as an Industrial Biotechnology Host. Plant J..

[175] Giordano M., Wang Q. (2017). Microalgae for Industrial Purposes. Biomass and Green Chemistry.

[176] Anderson M.S., Muff T.J., Georgianna D.R., Mayfield S.P. (2017). Towards a Synthetic Nuclear Transcription System in Green Algae: Characterization of *Chlamydomonas Reinhardtii* Nuclear Transcription Factors and Identification of Targeted Promoters. Algal Res..

[177] Barrera D.J., Mayfield S.P. (2013). High-Value Recombinant Protein Production in Microalgae. Handbook of Microalgal Culture: Biotechnology and Applied Phycology.

[178] Fields F.J., Ostrand J.T., Mayfield S.P. (2018). Fed-Batch Mixotrophic Cultivation of *Chlamydomonas Reinhardtii* for High-Density Cultures. Algal Res..

[179] Mayfield S.P., Manuell A.L., Chen S., Wu J., Tran M., Siefker D., Muto M., Marin-Navarro J. (2007). Chlamydomonas Reinhardtii Chloroplasts as Protein Factories. Curr. Opin. Biotechnol..

[180] Franklin S., Ngo B., Efuet E., Mayfield S.P., Franklin S., Ngo B., Efuet E., Mayfield S.P. (2002). Development of a GFP Reporter Gene for Chlamydomonas Reinhardtii Chloroplast. Plant J..

[181] Loera-Quezada M.M., Leyva-González M.A., Velázquez-Juárez G., Sanchez-Calderón L., Do Nascimento M., López-Arredondo D., Herrera-Estrella L. (2016). A Novel Genetic Engineering Platform for the Effective Management of Biological Contaminants for the Production of Microalgae. Plant Biotechnol. J..

[182] Takouridis S.J., Tribe D.E., Gras S.L., Martin G.J.O. (2015). The Selective Breeding of the Freshwater Microalga *Chlamydomonas Reinhardtii* for Growth in Salinity. Bioresour. Technol..

[183] Chen M., Li L.Z.S., Chang S., Wang W., Zhang Z., Wang J., Zhao G., Qi L., Xu W. (2014). Characterization of Cell Growth and Photobiological H2 Production of Chlamydomonas Reinhardtii in ASSF Industry Wastewater. Int. J. Hydrogen Energy.

[184] Tran M., Chang J.T., Hansen J., Mayfield M., Mayfield S.P., Rasala B.A., Wang X. (2020). Inventors; Triton Algae Innovations, Assignee. Method of Treating Alimentary Canal Conditions. Method of Treating Alimentary Canal Conditions. U.S. Patent.

[185] Koller M., Muhr A., Braunegg G. (2014). Microalgae as Versatile Cellular Factories for Valued Products. Algal Res..

[186] Pulz O., Gross W. (2004). Valuable Products from Biotechnology of Microalgae. Appl. Microbiol. Biotechnol..

[187] Gunasekaran B., Gothandam K.M. (2020). A review on edible vaccines and their prospects. Braz. J. Med. Biol. Res..

